# Timing of follow-up visits after hospital discharge for COPD: Application of a new method

**DOI:** 10.1371/journal.pone.0302681

**Published:** 2024-07-10

**Authors:** Lili Jiang, Peter C. Austin, Walter P. Wodchis, Tara Kiran, Jun Guan, Andrea S. Gershon

**Affiliations:** 1 Sunnybrook Health Sciences Centre, Toronto, Ontario, Canada; 2 ICES, Toronto, Ontario, Canada; 3 Institute of Health Policy, Management and Evaluation, University of Toronto, Toronto, Ontario, Canada; 4 Institute for Better Health; Trillium Health Partners, Mississauga, Ontario, Canada; 5 Department of Family and Community Medicine, University of Toronto, Toronto, Ontario, Canada; 6 MAP Centre for Urban Health Solutions and the Department of Family and Community Medicine, St. Michael’s Hospital, Toronto, Ontario, Canada; 7 Department of Medicine, University of Toronto, Toronto, Ontario, Canada; King Saud University Medical City, SAUDI ARABIA

## Abstract

**Rationale:**

A common strategy to reduce COPD readmissions is to encourage patient follow-up with a physician within 1 to 2 weeks of discharge, yet evidence confirming its benefit is lacking. We used a new study design called target randomized trial emulation to determine the impact of follow-up visit timing on patient outcomes.

**Methods:**

All Ontario residents aged 35 or older discharged from a COPD hospitalization were identified using health administrative data and randomly assigned to those who received and did not receive physician visit follow-up by within seven days. They were followed to all-cause emergency department visits, readmissions or death. Targeted randomized trial emulation was used to adjust for differences between the groups. COPD emergency department visits, readmissions or death was also considered.

**Results:**

There were 94,034 patients hospitalized with COPD, of whom 73.5% had a physician visit within 30 days of discharge. Adjusted hazard ratio for all-cause readmission, emergency department visits or death for people with a visit within seven days post discharge was 1.03 (95% Confidence Interval [CI]: 1.01–1.05) and remained around 1 for subsequent days; adjusted hazard ratio for the composite COPD events was 0.97 (95% CI 0.95–1.00) and remained significantly lower than 1 for subsequent days.

**Conclusion:**

While a physician visit after discharge was found to reduce COPD events, a specific time period when a physician visit was most beneficial was not found. This suggests that follow-up visits should not occur at a predetermined time but be based on factors such as anticipated medical need.

## Introduction

Chronic obstructive pulmonary disease is a leading cause of mortality and morbidity globally [1], with a hospitalization rate of 28.8 per 1000 person-years [2]. COPD hospitalizations are often not isolated events, as readmissions within 30 days occur for about 20% of people—the highest rate of readmission among medical patients [3,4]. A common strategy to reduce readmission is to recommend that all patients receive a physician follow-up visit within 1 to 2 weeks of discharge [5–7]. This strategy, however, uses time sensitive resources, and there is limited evidence supporting it. While it seems intuitive that a visit shortly after discharge would be beneficial, confirming that a visit within 1 week is better than a visit within 4 weeks would help justify use of resources that could be used for other patients. Previous observational study of follow-up visits effectiveness have been mixed and limited—commonly by lack of adjustment for confounders [6,8–15].

A randomized controlled trial (RCT) is the best study design to determine causation because, on average, it eliminates systematic differences in both measured and unmeasured baseline covariates between comparison groups. To the best of our knowledge, however, no RCT has studied timing of follow-up visits—likely because it would be difficult to convince patients and their health care providers to enter a study where they could be randomized to a delayed care group. RCTs are also subject to other limitations, such as reduced generalizability and real world relevance. Thus, evidence on the timing of post-discharge visits will likely be provided by observational studies. However, typical observational studies of the timing of follow-up visits are subject to confounding by indication-, survivor-treatment- and immortal-time bias. Specifically, in typical practice, individuals at higher risk for readmissions are also more likely to receive earlier follow-up care. Subjects who die early have less time to receive early follow-up, making early follow-up appear beneficial, and similarly, subjects who receive late follow-up have, by definition, not died prior to that late follow-up, making late follow-up appear beneficial.

Hernán have developed a method called ‘target randomized trial emulation’[16] that avoids the described biases and allows one to use observational data to estimate the effects of timing of treatment on outcomes. It does so by mimicking a RCT by randomizing subjects to receive or not receive a certain medical practice (an ‘intervention’, that could be medication, a procedure, a test, or in this case a follow-up visit within a certain time) at baseline and then follow them forward in time. Then, because patients’ exposure (i.e., timing of follow-up) is already determined at the start of follow-up, it uses statistical methods to account for people not staying in the group they were randomized to—either because people randomized to the ‘intervention’ group did not receive the ‘intervention’, or vice versa. While this approach has been used in many areas of medicine, to the best of our knowledge it has not been applied to respiratory disease [17,18].

Therefore, to determine the impact of early physician follow-up on readmission and survival, to explore the optimal timing of that follow-up, and to develop an exciting new study design for the study of respiratory disease, we used ‘target randomized trial emulation’ to study all patients discharged from COPD hospitalization in Ontario, Canada.

## Methods

### Study design and setting

We conducted a retrospective longitudinal population-based study of residents aged 35 or older using health administrative data from Ontario, Canada between April 1, 2004 and March 31, 2016. The use of the data in this project was authorized under section 45 of Ontario’s Personal Health Information Protection Act (PHIPA) and does not require review by a Research Ethics Board, nor informed consent (see below).

### Data sources

Ontario is the largest province of Canada with an ethnically diverse population of about 14 million. All physician and hospital expenses are paid for by the government of Ontario, the single payer for virtually all the population. Three government health administrative databases were used for this study. The Registered Persons Database contains basic demographic information, including sex, date of birth and date of death (if applicable), for all Ontario residents. The Ontario Health Insurance Plan Physician Claims database contains information on all physician visits provided by fee-for-service physicians and “shadow-billings” for physicians paid under alternate payment plans. The Canadian Institute of Health Information Discharge Abstract database contains information on all acute care hospitalizations including hospital readmissions. We also used the ICES Physician Database to identify physicians’ specialties. These datasets were linked on an individual level using unique encoded identifiers and analyzed at ICES. This was to provide a complete health services profile for each resident of Ontario. ICES, an independent, non-profit research institute whose legal status under Ontario’s health information privacy law allows it to collect and analyze health care and demographic data, without consent, for health system evaluation and improvement. All direct personal identifiers are removed from the data before being provided to researchers, making it effectively fully anonymized.

### Study population

Our study population included all insured Ontario residents aged 35 or older discharged from a hospitalization where COPD was a contributing diagnosis between April 1, 2004 and March 31, 2016. COPD was identified with International Classification of Diseases, 10th Revision, Canada (ICD-10-CA) codes J41, J42, J43, J44. The hospital discharge date was the index date.

Patients who were ineligible for the Ontario Health Insurance plan, had a COPD (emergent or elective) hospital admission in the previous year and/or had had lung volume reduction surgery or lung transplantation were excluded from the study. Those who had a hospital admission in the previous year were excluded because people with more frequent hospitalizations were more likely to have higher care needs. We also excluded patients who received palliative services because they were likely to have different goals of care and be less likely to be readmitted. Patients with inpatient length of stay greater than 21 days were excluded because they were more likely to have complicated hospital courses that would make close follow-up necessary. Finally, for patients with multiple eligible admissions during the study period, one was randomly chosen to avoid selection bias due to hospitalization order and to capture all severities of disease.

### Main exposure

The main exposure was an ambulatory care visit with any type of physician within 30 days of hospital discharge. In secondary analyses, we examined ambulatory care visits with pulmonologist, as they would have more expertise and experience taking care of people with COPD.

### Outcomes

Patients were followed for 180 days after hospital discharge until the composite primary outcome of all-cause readmission, unscheduled ED visits, mortality or censoring occurred. All-cause readmissions were considered because they are of interest to patients, health care providers and policy makers [19]. We then looked at COPD readmission, unscheduled ED visits or mortality. A composite was chosen because readmissions and unscheduled ED visits are often on the causal pathway to death, they are all outcomes that physician follow-up visits are meant to prevent, and because all are outcomes of importance to patients, physicians and decision makers. The time period of 180 days was chosen to determine if physician follow-up had an enduring impact, but shorter periods of 30, 60 and 90 days were also examined. Secondary outcomes included all-cause hospital readmissions and ED visits alone.

### Baseline characteristics

We considered a wide variety of baseline characteristics including demographic features such as age, sex, and socioeconomic status; comorbidities such as heart failure, dementia and lung cancer; COPD factors such as duration, previous hospitalizations, and specialist care; and other health care factors such as influenza vaccination and continuity of care. For some comorbidities like asthma, heart failure and hypertension the look back period considered all data available since 1992. For other comorbidities like cor pulmonale, pulmonary embolism, chronic kidney disease, dementia, lung cancer and osteoporosis the look back period was 5 years. Socioeconomic status was determined ecologically by using census data and patients’ postal code. We used aggregate diagnostic groups from the Johns Hopkins ACG® System Version 10.0 to categorize overall level of comorbidity. We obtained immigration data from the Immigration, Refugees and Citizenship Canada Permanent Resident Database.

### Statistical analysis

Descriptive statistics were used to summarize baseline characteristics of patients with and without a physician visit within 30 days post discharge.

We followed the four steps described by Hernán to emulate an RCT to estimate the effect of timing of physician follow-up visit on the outcomes [16]. First, without considering patients’ actual physician follow-up time, we randomly assigned each patient to one of to two groups: those that received follow-up by a certain day and those who did not receive follow up by that day—for example, patients who received a follow up visit by day 7 and those who did not receive a follow up visit by day 7 to create two groups of patients most likely balanced on all baseline characteristics. We then followed each subject and censored them at the time when their assigned group became inconsistent with the actual follow-up that they received. Thus, a patient randomly assigned to follow-up by day 7 was censored at day 7 if they had not received follow-up as of that time. Similarly, a patient randomly assigned to no follow-up by day 7 was censored at the time of a follow-up visit, if this was received on or before day 7. Third, this censoring could have been informative and introduced bias because it provided information on a patient’s prognosis, so that those who were censored were no longer representative of those who were not censored. To address this, we used stabilized inverse probability censoring weighting (IPCW) to balance those who were censored with those who were not censored. Specifically, weights were estimated using a traditional Cox regression model and all the variables mentioned, including age, sex, neighbourhood Income quintile, rurality, residence on long term care, immigration status, asthma, diabetes, heart failure, hypertension, lung cancer, non-lung cancer, cor pulmonale, pulmonary embolism, other chronic respiratory disease, chronic kidney disease, osteoporosis, dementia, mental health condition, overall level of comorbidity, duration of COPD, previous COPD hospitalization, previous COPD ED visits, receipt of spirometry, specialist care for COPD, number of primary care physician visits in the previous year, influenza vaccination and continuity of care with the outcome of interest being censoring. We then computed the probability of censoring using an unadjusted model (i.e., a model with no covariates) and a fully adjusted model (with all covariates included). The stabilized IPC weights were derived by dividing the former probability by the latter probability. Finally, outcomes of the group assigned to receive physician follow-up within that certain number of days (ie. within 7 days) was compared to the group assigned to not receive follow-up by that number of days using a univariate Cox model, weighted by the IPCW weights. This gave adjusted estimated hazard ratios (HR) for the effect of follow-up by the given day (ie. day 7), with people not having received follow-up by that day as the reference group. To understand the stability of the randomization procedure, we replicated these steps ten times, each with a different randomization. A complete case analysis was done.

To identify optimal timing of post discharge physician visit, the above steps were repeated 23 times, each time changing number of days that defined the groups were assigned to. Thus 7 days, 8 days, all the way to 30 days were examined. We did not examine follow-up earlier than 7 days because outcomes from such visits were more likely related to the previous hospital admission, rather than a physician visit afterwards.

All statistical tests used p<0.05 as the level of significance and were performed using the R statistical programing language (version 3.0.2) and SAS version 9.4 (SAS Institute, Cary, NC, USA).

### Additional analysis

Similar analysis was done for all-cause hospital readmission and ED visits. Death was considered a competing risk using Fine-Gray subdistribution hazard models [20].

The primary analysis was performed with different lengths of follow-up of 30 days, 60 days or 90 days. It was also repeated with the main exposure being a pulmonologist visit.

## Results

**Fig 1:**
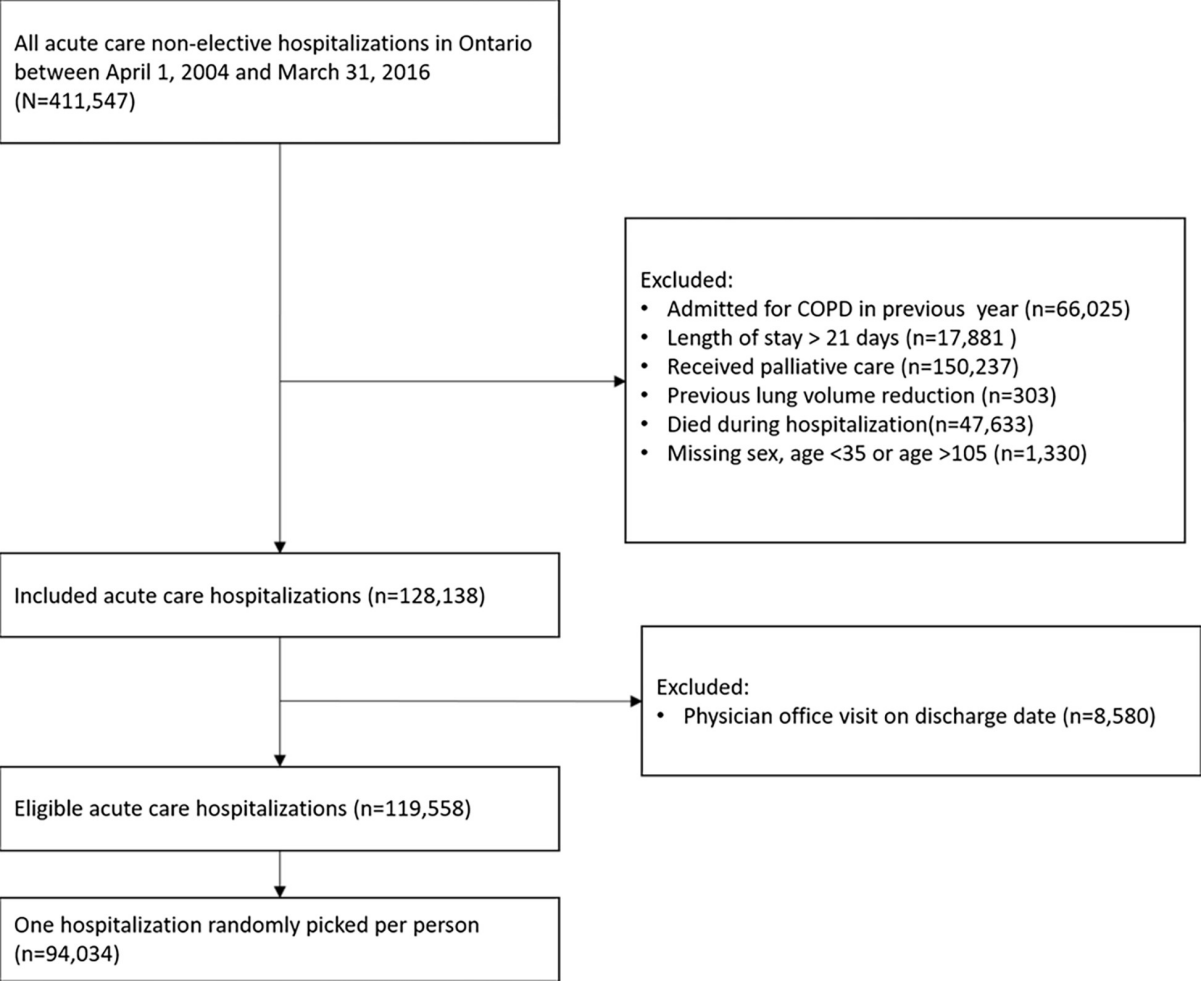
Study flow chart.

**Fig 2:**
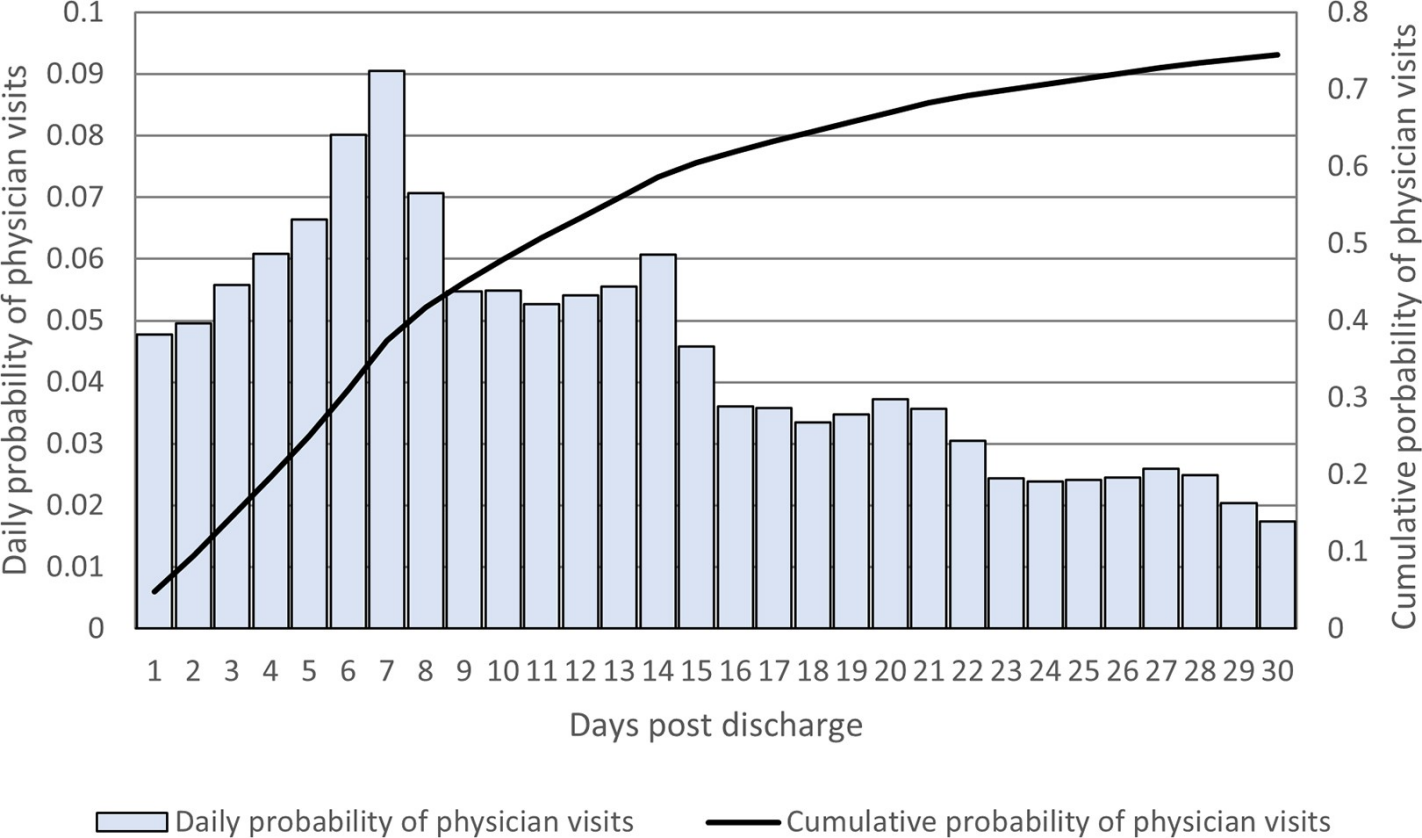
Distribution of physician visits among discharged COPD patients.

There were 411,547 non-elective hospitalizations for COPD with discharge dates between April 1, 2004 and March 31, 2016. After applying exclusions (**Fig 1**), 94,034 hospitalized patients were included in the final analysis. Approximately 73.5% had a visit with a physician within 30 days. Most patients had a physician visit soon after discharge, with the median visit occurring 11 days after discharge (**Fig 2**). Only 7.7% of people were seen by a pulmonologist within 30 days and 3.3% were seen by a pulmonologist before any other type of physician.

Patients who had a physician visit within 30 days were older, less like to live in a rural area and more likely to be of higher economic status. They also had higher rates of ambulatory care visits, spirometry testing and influenza vaccination in the year prior to the index date (**Table 1).**

**Table 1:** Baseline characteristics for study population by having any physician office visit within 30 days post discharge.

Characteristic		Total	Any physician office visit within 30 days *before* simulated randomization			Any physician office visit within 30 days *after* simulated randomization		
			No	Yes	Standardized Difference	No	Yes	Standardized Difference
N		94,034	24,919	69,115		46984	47050	
Male Sex, n (%)		47,066 (50.1)	12,602 (50.6)	34,464 (49.9)	0.014	23449 (49.9)	23617 (50.2)	0.006
Age, years, Median (IQR)		74.0 (64.0–82.0)	73.0 (63.0–82.0)	74.0 (64.0–82.0)	0.032	74.0 (64.0–82.0)	74.0 (64.0–82.0)	0.002
Neighbourhood Income quintile, n (%)					0.111			0.004
	1 (lowest)	26,475 (28.2)	7,890 (31.7)	18,585 (26.9)		13211 (28.1)	13264 (28.2)	
	2	20,946 (22.3)	5,468 (21.9)	15,478 (22.4)		10450 (22.2)	10496 (22.3)	
	3	18,088 (19.2)	4,619 (18.5)	13,469 (19.5)		9050 (19.3)	9038 (19.2)	
	4	15,674 (16.7)	3,776 (15.2)	11,898 (17.2)		7862 (16.7)	7812 (16.6)	
	5(highest)	12,851 (13.6)	3,166 (12.7)	9,685 (14.0)		6411 (13.6)	6440 (13.7)	
Rurality, n (%)^1^		18,729 (19.9)	6,049 (24.3)	12,680 (18.4)	0.145	9322 (19.8)	9407 (20.0)	0.004
Resident in long-term care		7,774 (8.3)	1,679 (6.7)	6,095 (8.8)	0.078	3854 (8.2)	3920 (8.3)	0.005
Immigrant, n (%)		1,230 (1.3)	276 (1.1)	954 (1.4)	0.025	636 (1.4)	594 (1.3)	0.008
COPD duration, years, n (%)					0.021			0.005
	< 1yr	27,007 (28.7)	7,330 (29.4)	19,677 (28.47)		13450 (28.6)	13557 (28.8)	
	1-5yr	15,563 (16.6)	4,062 (16.3)	11,501 (16.6)		7758 (16.5)	7805 (16.6)	
	5+yr	51,464 (54.7)	13,527 (54.3)	37,937 (54.9)		25776 (54.9)	25688 (54.6)	
Spirometry in previous year, n (%)		25,916 (27.6)	5,900 (23.7)	20,016 (28.9)	0.12	13080 (27.8)	12836 (27.3)	0.012
Number of primary care physician visits in previous year, Median (IQR)		7.0 (3.0–12.0)	5.0 (2.0–9.0)	8.0 (4.0–13.0)	0.496	7.0 [3.0, 12.0]	7.0 [3.0, 12.0]	0.001
Influenza vaccine in previous year, n (%)		40,699 (43.3)	8,612 (34.6)	32,087 (46.4)	0.244	20400 (43.4)	20299 (43.1)	0.006
Mental health condition, n (%)		9,079 (9.7)	2,470 (9.9)	6,609 (9.6)	0.012	4571 (9.7)	4508 (9.6)	0.005
Level of comorbidity, n (%)					0.159			0.009
	low	10,627 (11.3)	3,724 (14.9)	6,903 (10.0)		5266 (11.2)	5361 (11.4)	
	moderate	31,411 (33.4)	8,390 (33.7)	23,021 (33.3)		15639 (33.3)	15772 (33.5)	
	high	51,996 (55.3)	12,805 (51.4)	39,191 (56.7)		26079 (55.5)	25917 (55.1)	
Asthma, n (%)		39,621 (42.1)	9,984 (40.1)	29,637 (42.9)	0.057	19800 (42.1)	19821 (42.1)	<0.001
Diabetes, n (%)		33,037 (35.1)	8,538 (34.3)	24,499 (35.5)	0.025	16555 (35.2)	16482 (35.0)	0.004
Heart failure, n (%)		36,639 (39.0)	9,591 (38.5)	27,048 (39.1)	0.013	18195 (38.7)	18444 (39.2)	0.01
Hypertension, n (%)		68,110 (72.4)	17,184 (69.0)	50,926 (73.7)	0.105	34045 (72.5)	34065 (72.4)	0.001
Non-lung Cancer, n (%)		13,869 (14.8)	3,363 (13.5)	10,506 (15.2)	0.049	7033 (15.0)	6836 (14.5)	0.012
Pulmonary embolism in previous 5 years, n (%)		473 (0.5)	114 (0.5)	359 (0.5)	0.009	243 (0.5)	230 (0.5)	0.004
Cor pulmonale, n (%)		230 (0.2)	68 (0.3)	162 (0.2)	0.008	121 (0.3)	109 (0.2)	0.005
Chronic Kidney Disease, n (%)		5,012 (5.3)	1,575 (6.3)	3,437 (5.0)	0.058	2508 (5.3)	2504 (5.3)	0.001
Dementia, n (%)		15,089 (16.1)	4,001 (16.1)	11,088 (16.0)	<0.001	7473 (15.9)	7616 (16.2)	0.008
Lung Cancer, n (%)		1,493 (1.6)	376 (1.5)	1,117 (1.6)	0.009	766 (1.6)	727 (1.5)	0.007
Other Chronic respiratory disease, n (%)		7,344 (7.8)	2,031 (8.2)	5,313 (7.7)	0.017	3700 (7.9)	3644 (7.7)	0.005
Osteoporosis, n (%)		8,088 (8.6)	2,349 (9.4)	5,739 (8.3)	0.04	4115 (8.8)	3973 (8.4)	0.011
Previous COPD hospitalization within 5 years, n (%)		17,633 (18.8)	5,147 (20.7)	12,486 (18.1)	0.066	8874 (18.9)	8759 (18.6)	0.007
Previous hospitalization for cardiovascular diseases^2^ within 5 years, n (%)		31,155 (33.1)	8,333 (33.4)	22,822 (33.0)	0.009	15524 (33.0)	15631 (33.2)	0.004
Previous COPD ED visit within 5 years, n (%)		23,490 (25.0)	6,929 (27.8)	16,561 (24.0)	0.088	11743 (25.0)	11747 (25.0)	0.001
Previous all-cause ED visits within 5 years, n (%)		76,808 (81.7)	20,787 (83.4)	56,021 (81.1)	0.062	38444 (81.8)	38364 (81.5)	0.007
Continuity of care–Bice method, Median (IQR)^3^		0.74 (0.46–0.94)	0.67 (0.36–0.92)	0.76 (0.49–0.94)	0.21	0.74 [0.46, 0.94]	0.74 [0.46, 0.94]	0.004

COPD, chronic obstructive pulmonary disease; ED, emergency department; IQR, interquartile range.

^1^Data was missing for 0.04%.

^2^Including ischemic heart disease, heart failure, stroke and dysrhythmias.

^3^Continuity of care measured using the Bice method. The index ranges from 0 (each visit involves a different clinician than all other visits) to 1 (all visits were billed by a single clinician), where higher scores represent greater continuity [21].

Fifty percent of patients who had a physician visit within 30 days experienced the composite outcome of all-cause readmission, unscheduled ED visit or mortality at the end of 180 days compared to 71.2% of people who did not have a physician visit.

After simulated randomization, baseline characteristics were balanced between people who did and did not see physician on each day with all standardized differences being less than 0.10.

**Fig 3:**
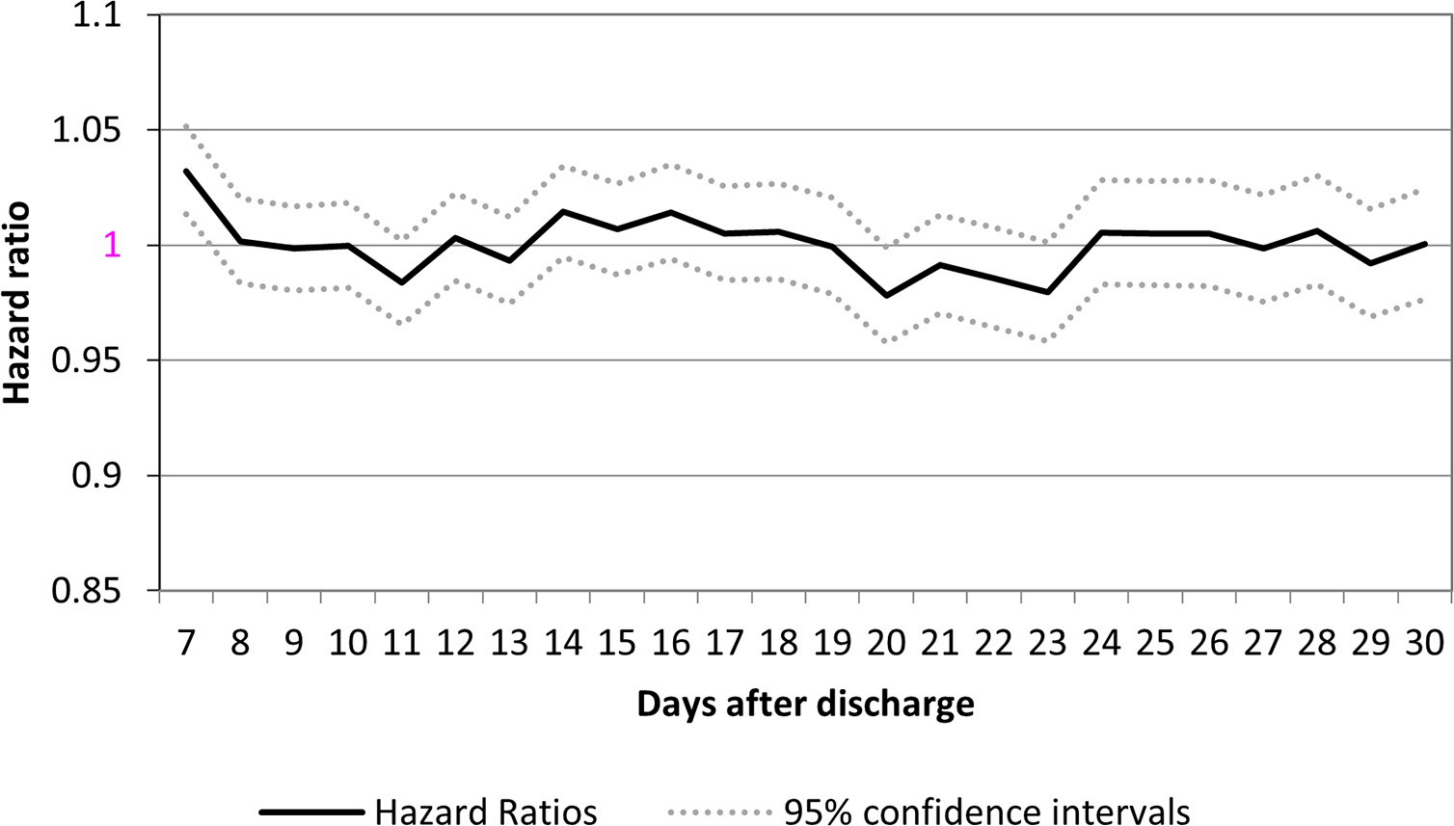
Hazard ratios of all-cause emergency department visits, hospital readmissions or death at 180 days in individuals receiving a physician visit compared with individuals not receiving a physician visit on days 7 through 30 post discharge.

**Fig 3** displays the hazard ratios of the primary outcome in patients having a physician visit compared to those not having a physician visit by days 7 to 30 post discharge. The HR remained around 1. The HR was 1.03 (95% Confidence Interval [CI]: 1.01–1.05) for visits within 7 days of discharge and continued to hover around 1 for visits within 8 days onwards. When the analysis was repeated (with different randomizations) ten times, the results did not change significantly.

**Fig 4:**
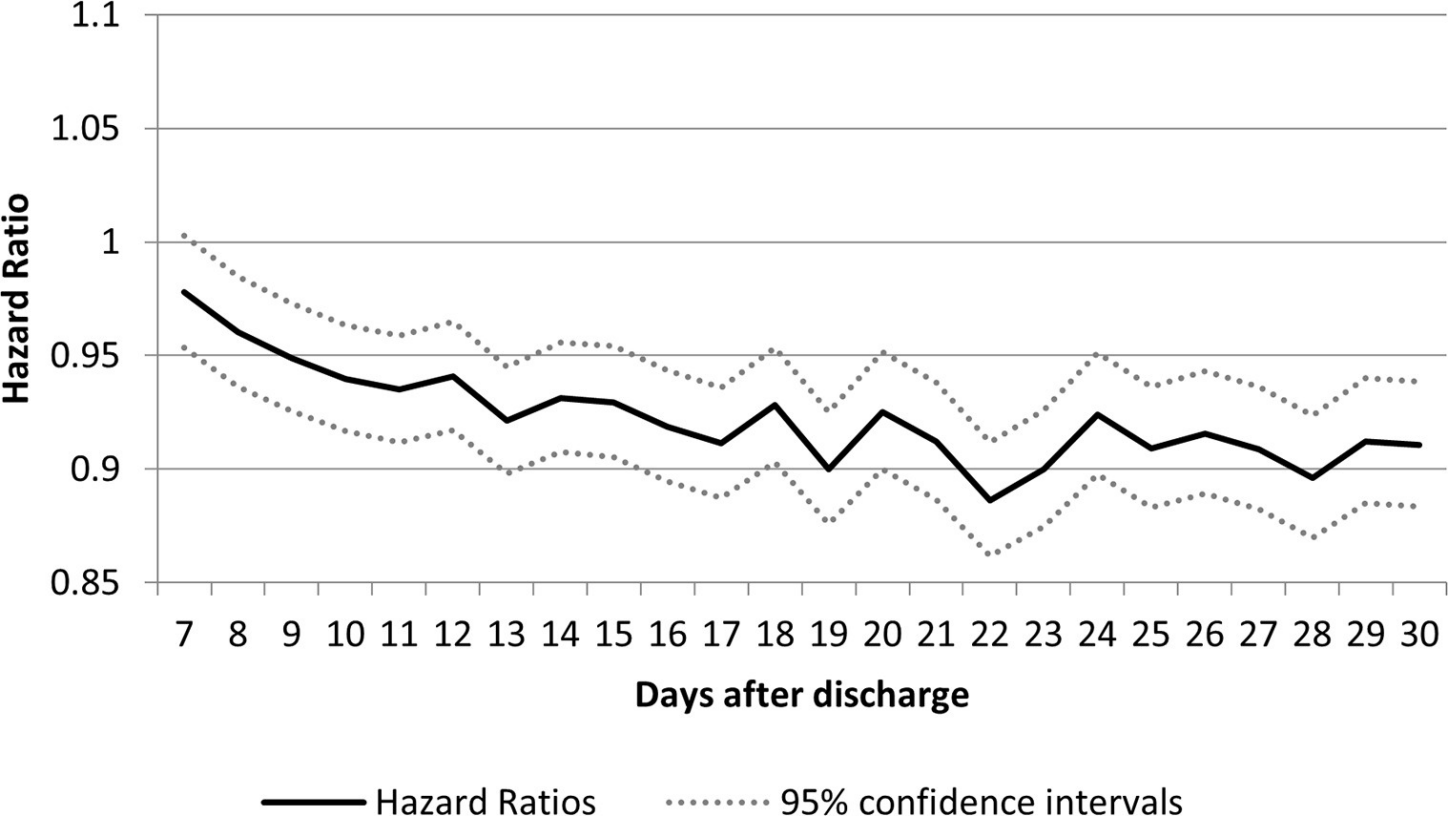
Hazard ratios of COPD emergency department visits, hospital readmissions or death at 180 days in individuals receiving a physician visit compared with individuals not receiving a physician visit on days 7 through 30 post discharge.

When the composite outcome of COPD readmission, COPD ED visits and all-cause mortality was considered (**Fig 4**), the HR for within 7 days was 0.98 (95% CI:0.96–1.00). From days 8 to 30 it remained significantly below 1 with a low of 0.89 (95% CI: 0.86–0.91) on day 22.

**Fig 5:**
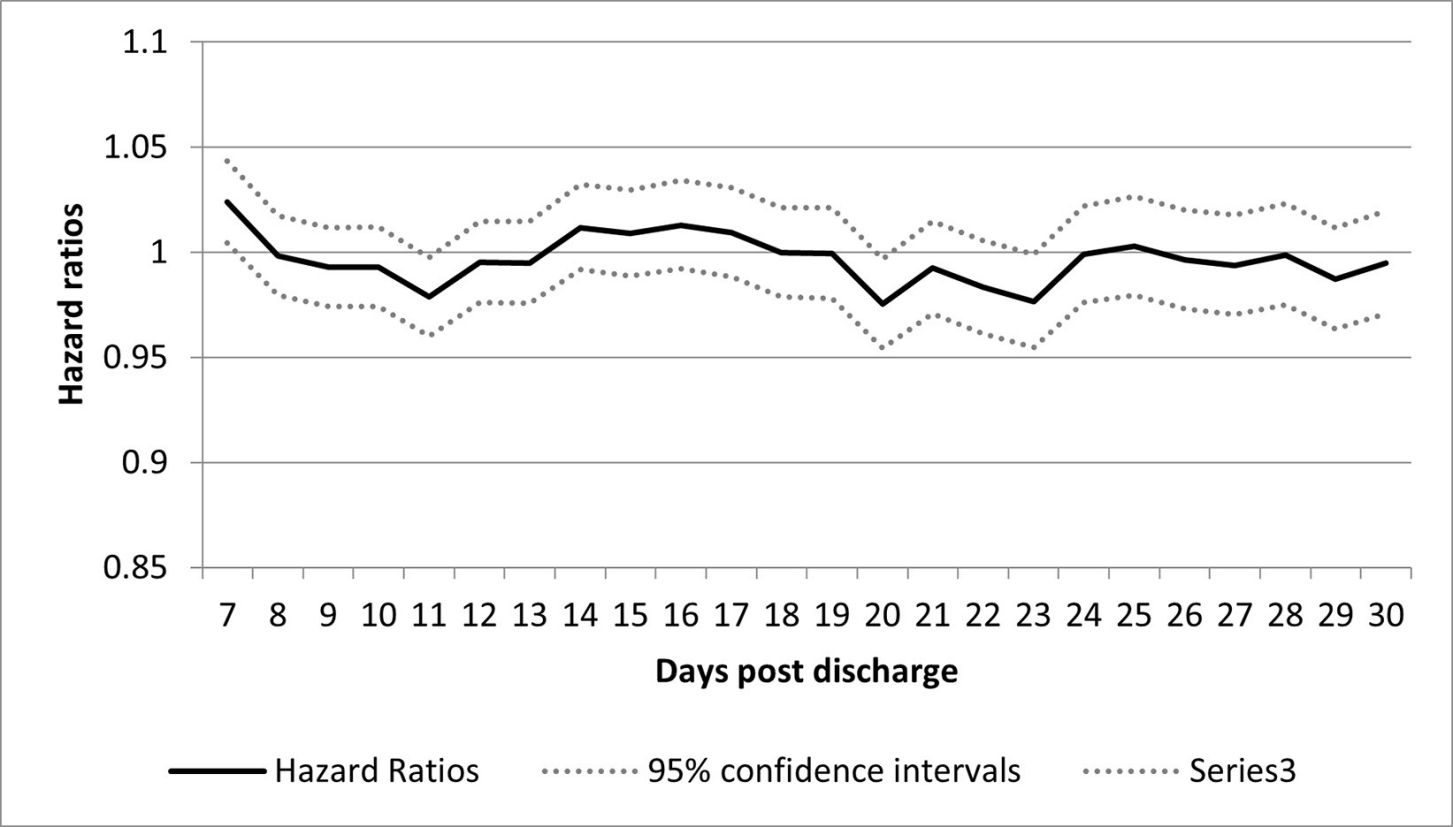
Hazard ratios of emergency department visit and hospital readmissions with death as a competing risk at 180 days in individuals receiving a physician visit compared with individuals not receiving a physician visit on days 7 through 30 post discharge.

Analysis of all-cause hospital readmission and ED visits considering the competing risk of death revealed similar findings as those seen with the primary composite outcome (**Fig 5**).

When the primary analysis was repeated with the follow-up period shortened to 30, 60 and 90 days, HRs still hovered around 1; however, there was a suggestion that HRs were lower between 8 and 13 days than at other times. Similar findings to the main analysis were also found when the exposure was changed from all type of physician visit to pulmonologist visit only. **(S1 and S2 Figs)**.

## Discussion

We conducted a population-based real-world study using health administrative data to emulate a randomized clinical trial to examine the association between early physician follow-up within days 7 through to 30 post discharge and all-cause ED visits, hospital readmissions or death within 180 days after discharge. Our findings were *not* suggestive of a specific period of time during which a physician visit after discharge would decrease the composite of emergency department visits, hospitalizations and death; however they did suggest that seeing a physician decreased COPD emergency department visits, hospitalizations and death. We believe that we did not find such a time period because, in our real-world, observational study, discharged patients were appropriately scheduled follow-up appointments based on factors like medical need. In other words, patients and health care providers in Ontario were good at knowing appropriate timing of follow-up. This is consistent with findings of a qualitative study of COPD and heart failure patients examining the role of physician visits post hospital discharge [22].

Our target randomized trial had many strengths that previous observational studies did not have. To begin, through randomization simulation (that was repeated multiple times), confounders such as age, sex, socioeconomic status, and disease severity were balanced between people who were and were not assigned to have early physician follow-up, minimizing indication and survivor bias. Further, through randomization, unmeasured confounders were also balanced. As in any RCT, we would expect that, on average, measured and unmeasured baseline confounders will be balanced. In our study, this is simply a consequence of randomly assigning subjects to one of the two groups.

Second, we were able to use real world, population-based data and thus include people with COPD of various ages, socioeconomic status, location of residence, comorbidity and other characteristics, making the findings highly generalizable. Finally, using this approach we were able to avoid immortal time bias as the exposure was assigned at the beginning of the follow-up for both groups. Immortal time bias occurs when the date of exposure follows the date patients enter into the study and the study design does not account for the fact that it is impossible for death to occur between the two.

We are only aware of one previous study that examined the relationship between timing of physician follow-up visit and readmissions. In that study COPD was one of several conditions examined [15]. It also did not reveal a specific time period when a physician visit after discharge would be most beneficial, however, it was limited by immortal time bias.

There are other explanations for not finding a specific time period when physician visits post-discharge would be most beneficial. One possibility is that early physician follow-up in the month following discharge does not reduce all-cause ED visit, hospital readmission or deaths. We found that a visit within a certain number of days was not better than not having a visit within that number of days—findings that are consistent with previous observational studies that did not find an association between physician visits within 30 days and readmissions [11,12]. A second explanation is that seeing a physician post-discharge is on the causal pathway to ED visit, hospital readmission and death making the physician visits and readmissions highly correlated and hazard ratios around 1 expected. Finally, a third explanation is that early physician follow-up might have decreased COPD readmissions for some patients, but improved communication and awareness of other types of medical problems in others leading to increasing readmissions for them [23]. The net results would be no apparent improvement. Our secondary analysis showing early physician follow-up was associated with a modest reduction in COPD-specific outcomes supports this hypothesis.

Our findings were not consistent with other studies that reported that patients receiving a physician follow-up visit had a lower risk of 30-day readmission [8–10]. Methodologic limitations such as no or little adjustment for potential confounders, as described above, and/or immortal time bias could explain the discrepancies.

We noted that the hazards for readmission were increased at 7 days. We hypothesize that this is because patients seen within one week were more likely to be unstable from the index hospitalization. Thus a physician visit at 7 days was be on the causal pathway to a patient being readmitted to hospital. We purposely started our study after 1 week of discharge to avoid this; however, possibly still caught some patients on this trajectory.

Our study has limitations that merit emphasis. First, while we emulated a randomized controlled trial through random assignment of treatment, thereby avoiding indication and survivor bias by creating exposure groups that were balanced at baseline, we needed to then censor people when their actual treatment became discordant with their randomly assigned treatment. This discordance could have provided information on their prognosis, so that those who were not censored at a given time were no longer representative of those who are censored (due to treatment discordance) at the given time. Failure to account for this informative censoring might have introduced bias into the estimates. To address this we used inverse probability of censoring weights. However, the validity of this approach assumed that we had all the variables necessary to do so. While we used many clinically relevant variables, it is conceivable that not all variables related to the censoring process were available to us. This could have cause unmeasured confounding. The effect of such unmeasured confounding, if it occurred, on the direction, magnitude or clinical significance of the results is not known. Second, we did not adjust for other post-discharge disease management interventions for example homecare, non-physician visits, or phone calls that were not captured in the health administrative data at the time of the study [24–26]. However, many of these were likely on the causal pathway to readmission, so accounting for them could have—at least in part—led to overadjustment. Third, we only focused on whether patients received a post discharge physician visit and the timing of the visit, we did not consider the quality or comprehensiveness of the care provided at the visit. We also did not capture patient preferences. Finally, all-cause mortality—part of our composite outcome—can be due to many things unrelated to COPD. We choose to include it because of its clinical relevance; however, we conducted an additional analysis with death as a competing risk. Neither our analysis with death as a part of the composite or death as a competing risk revealed a specific period of time during which a physician visit after discharge would be most beneficial, confirming our results were robust.

In summary, we conducted a population study of people with COPD using a novel statistical method that emulated a randomized clinical trial to avoid several biases. We found that COPD ED visits, hospital readmission or death were reduced when patients visited a physician after discharge, however, we did not find specific timing of the physician visit that was most beneficial. These findings support a practice of having COPD patients follow up with a physician but not at a set time period after discharge. Optimal timing should likely be based on other factors such as anticipated medical need and patient preference.

## Supporting information

S1 Fig Hazard ratios of emergency department visit, hospital readmission or death at various times in individuals receiving a physician visit compared with individuals not receiving a physician visit on days 7 through 30 post discharge. (TIF)

S2 Fig Hazard ratios of emergency department visit, hospital readmission or death in individuals receiving a pulmonologist visit compared with individuals not receiving a pulmonologist visit on days 7 through 30 post discharge. (TIF)
