# Supplementary figures and images for: Timing of follow-up visits after hospital discharge for COPD: Application of a new method
Source: PLoS One. 2024 Jul 10;19(7):e0302681. doi: 10.1371/journal.pone.0302681 (PMC11236132; doi:10.1371/journal.pone.0302681)

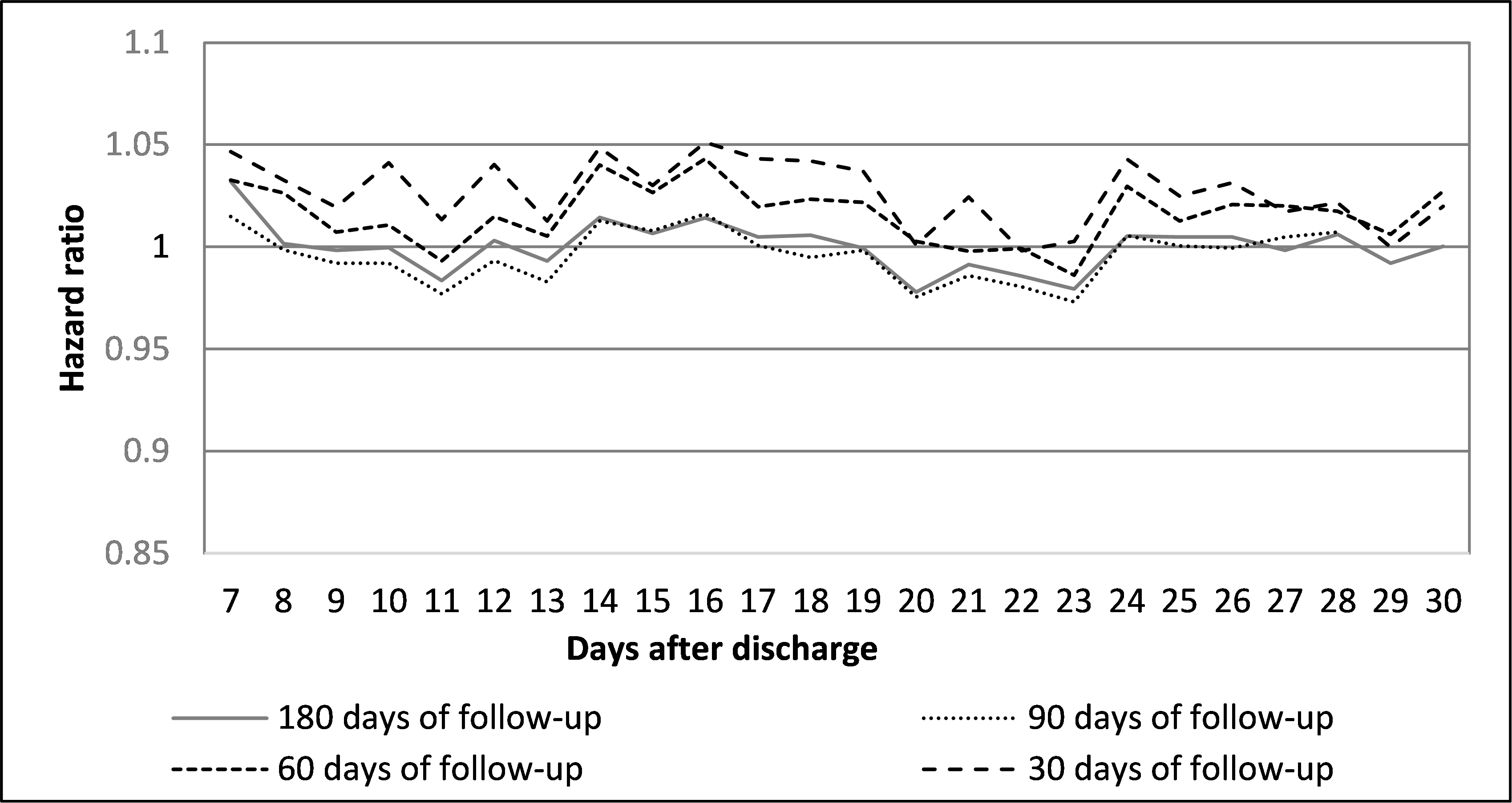

Supplement: S1 Fig — (TIF) [file pone.0302681.s001.tif]

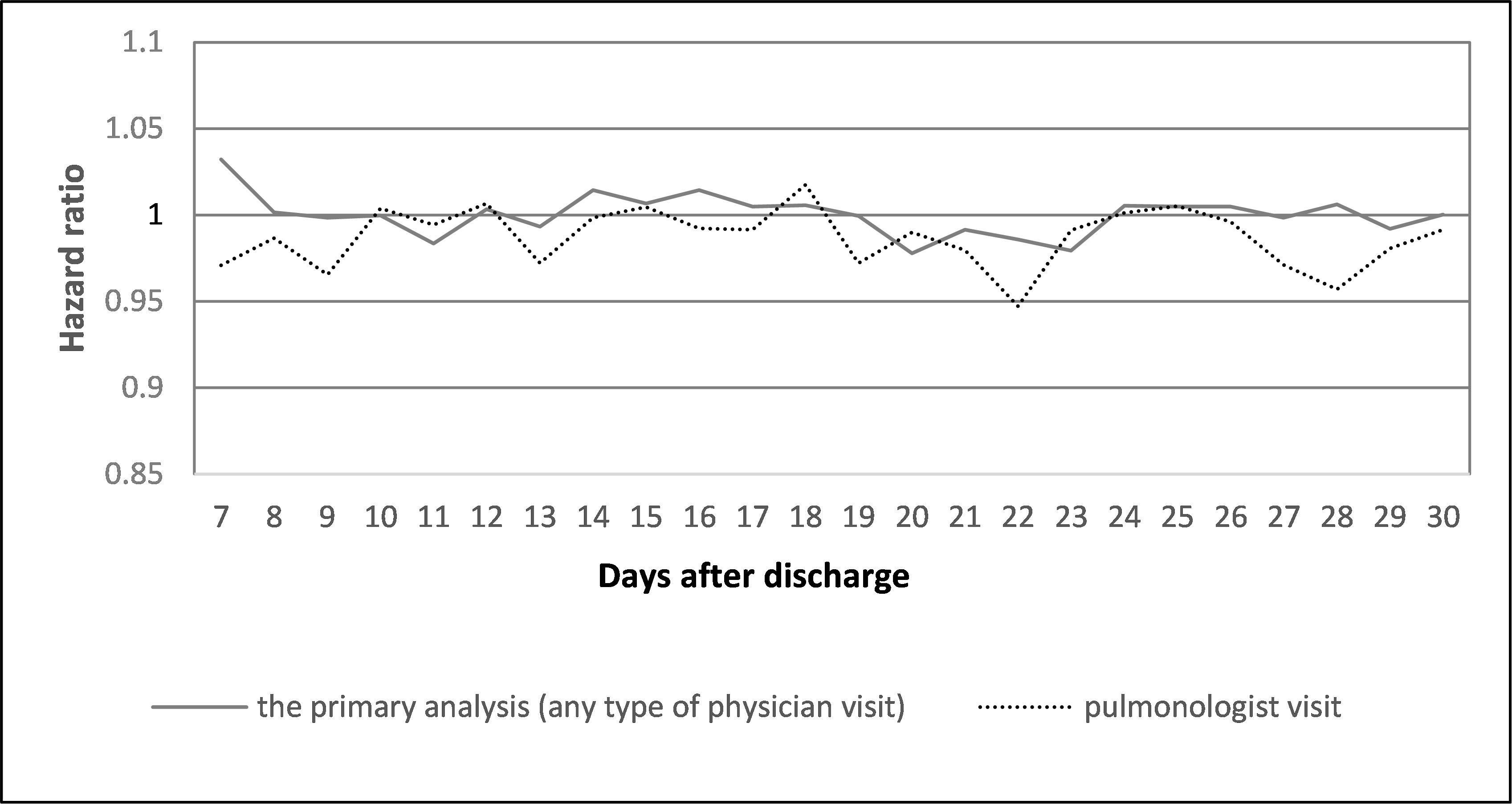

Supplement: S2 Fig — (TIF) [file pone.0302681.s002.tif]
